# The role of cholesterol metabolism and cholesterol transport in carcinogenesis: a review of scientific findings, relevant to future cancer therapeutics

**DOI:** 10.3389/fphar.2013.00119

**Published:** 2013-09-25

**Authors:** Pedro M. R. Cruz, Huanbiao Mo, Walter J. McConathy, Nirupama Sabnis, Andras G. Lacko

**Affiliations:** ^1^Department of Molecular Biology and Immunology, University of North Texas Health Science CenterFort Worth, TX, USA; ^2^Department of Nutrition and Food Sciences, Texas Woman’s UniversityDenton, TX, USA; ^3^LipoMedics Ltd. CoFort Worth, TX, USA

**Keywords:** cholesterol metabolism, lipoprotein transport, carcinogenesis, drug delivery system, SR-B1 receptor

## Abstract

While the unique metabolic activities of malignant tissues as potential targets for cancer therapeutics has been the subject of several recent reviews, the role of cholesterol metabolism in this context is yet to be fully explored. Cholesterol is an essential component of mammalian cell membranes as well as a precursor of bile acids and steroid hormones. The hypothesis that cancer cells need excess cholesterol and intermediates of the cholesterol biosynthesis pathway to maintain a high level of proliferation is well accepted, however the mechanisms by which malignant cells and tissues reprogram cholesterol synthesis, uptake and efflux are yet to be fully elucidated as potential therapeutic targets. High and low density plasma lipoproteins are the likely major suppliers of cholesterol to cancer cells and tumors, potentially via receptor mediated mechanisms. This review is primarily focused on the role(s) of lipoproteins in carcinogenesis, and their future roles as drug delivery vehicles for targeted cancer chemotherapy.

## INTRODUCTION

The term “cancer” designates a series of pathological changes characterized by deregulation of cell cycle and metabolism resulting in uncontrolled proliferation of cells (Teicher et al., 2012). While a wide range of metabolic pathways have been implicated in the process of carcinogenesis and (Hanahan and Weinberg, 2011) as potential targets for cancer prevention, diagnosis or treatment, relatively little has been written about the role(s) of cholesterol biosynthesis or metabolism in this regard. This hiatus of information is somewhat surprising as blood cholesterol levels [especially high density lipoprotein (HDL) levels] have been consistently shown to be lower in cancer patients (Ho et al., 1978; Vitols et al., 1984, 1990) and tumor membranes were found to be rich in cholesterol (Elegbede and Elson, 1986), suggesting that cholesterol utilization by malignant cells and tumors is an important feature of carcinogenesis and perhaps metastasis (Markel and Brook, 1994; Antalis et al., 2011). While the specific mechanisms involved in shifting normal cholesterol metabolism to the malignant phase is not yet understood, clinical and experimental findings suggest an apparent need of malignant cells and tissues for higher than normal amounts of cholesterol (Murtola et al., 2012) and intermediates of the cholesterol biosynthesis pathway (Mo and Elson, 2004). The purpose of this review is to provide an up to date assessment of the findings involving cholesterol metabolism and transport with a focus on opportunities for cancer therapeutics and tumor imaging.

## CHOLESTEROL AND CANCER

### CHOLESTEROL HOMEOSTASIS IN NORMAL CELLS AND TISSUES

Cholesterol plays an essential role in the stability and architecture of the plasma membrane and as a precursor for bile acid and steroid hormone synthesis in mammals (Simons and Ikonen, 2000). Under healthy homeostatic conditions, the circulating levels of cholesterol are regulated by a balance between local (cellular) synthesis, dietary cholesterol and removal of the excess cholesterol from peripheral tissues (Simons and Ikonen, 2000). The disturbance of this homeostatic state is known to be associated with cardiovascular diseases and is often attributed to dietary and lifestyle habits (Hu et al., 2012). Clinical and experimental evidence suggest that alterations in cholesterol metabolism may also have an important role in carcinogenesis and tumor development (Silvente-Poirot and Poirot, 2012b).

### CHOLESTEROL METABOLISM IN CANCER

Carcinogenesis is a complex process that involves massive reprograming of genetic information, signaling mechanisms, structural components, and energy metabolism (Hanahan and Weinberg, 2011; Biswas et al., 2012) of the cell. One could predict that this major transformation process is the consequence of the cancer cell’s increased metabolic requirements (Warburg, 1956) to sustain the tumor proliferation, migration and metastatic activities. Considering the importance and the efficient regulation of cholesterol metabolism and transport it is likely that these finely tuned mechanisms will become altered during the high velocity of cell division and membrane synthesis needed for carcinogenesis (Silvente-Poirot and Poirot, 2012b).

Fluctuations of cellular and blood cholesterol levels are controlled by a series of mechanisms that balance intracellular cholesterol synthesis with mechanisms of uptake and efflux (Simons and Ikonen, 2000; Solomon and Freeman, 2011). These mechanisms also coordinate intracellular cholesterol levels with progressive steps in the cell cycle (Simons and Ikonen, 2000; Pelton et al., 2012). The up-regulation of cholesterol biosynthesis and uptake and the down-regulation or impairment of cholesterol efflux from cells are considered to be consistent with carcinogenesis. Herein we provide a discussion of clinical and experimental data that reflect on these mechanisms.

#### Enhanced cholesterol biosynthesis

Cholesterol is synthesized via a cascade of enzymatic reactions known as the mevalonate pathway. This series of reactions is primarily regulated by a rate-limiting step involving the conversion of hydroxyl-methyl glutaryl-coenzyme A (HMG-CoA) into mevalonate. The rate limiting reduction of HMG-CoA to mevalonate is an important regulatory step in cholesterol synthesis that is commonly used as therapeutic target in hyperlipidemic disorders utilizing statin drugs. Studies of cancer cells reveal that cholesterol synthesis is enhanced, compared to untransformed cells (Larsson, 1996; Li et al., 2003; Mo and Elson, 2004; Clendening et al., 2010; Ginestier et al., 2012). Among the possible mechanisms that promote the upregulation of cellular cholesterol synthesis are: the abundant availability of precursors (acetyl-CoA), via glycolysis that also potentiates de novo fatty acid synthesis (Warburg, 1956; DeBerardinis et al., 2008; Biswas et al., 2012). Simultaneously, the activity of HMG-CoA reductase may also be increased due to increased transcriptional regulation [mediated by sterol regulatory element binding proteins (SREBPs)] or altered feedback control of HMG-CoA reductase (Chen and Kandutsch, 1978; Gregg and Sabine, 1982; Yachnin and Mannickarottu, 1984; Yachnin and Toub, 1984; Erickson and Cooper, 1988; Azrolan and Coleman, 1989; Llaverias et al., 2011). In human PC-3 and LNCaP prostate tumor cells, SREBP-2 activity had a strong correlation with cell viability (Krycer and Phan, 2012). Consistent with the above findings, the administration of mevalonate was shown to enhance tumor growth in mice carrying tumor xenograft (Duncan et al., 2004). Taken together these findings validate the importance of the mevalonate pathway in cancer metabolism and identify a target for potential novel therapeutic strategies via small molecules or gene targeting.

#### Inhibition of the mevalonate pathway

Inhibition of the mevalonate pathway commonly taken advantage during the treatment of dyslipidemic disorders. Statins are competitive inhibitors of HMG-CoA reductase, thus blocking the progression of the mevalonate pathway and limiting the downstream reactions and accumulation of the final products: cholesterol, isoprenoids, dolichol, ubiquinone, and isopentenyladenine (Clendening et al., 2010; Thurnher et al., 2012).

Available experimental evidence strongly suggests that the inhibition of the mevalonate pathway using statin drugs has an impact on oncogenic events such as cell division, tumor growth, and metastatic potential (Duncan et al., 2004; Clendening and Penn, 2012; Thurnher et al., 2012). Inhibition of the mevalonate pathway blocks the synthesis of isoprenoid molecules (farnesyl pyrophosphate and geranylgeranyl pyrophosphate) that facilitate post-translational modification and activate Ras, Rac, and Rho GTPases molecules contributing to tumor proliferation (Zhuang et al., 2005; Solomon et al., 2009; Gorin et al., 2012). While statin therapy blocks the intracellular synthesis of cholesterol, it also alters the cholesterol content of tumor cell membranes, interfering with key signaling pathways (Zhuang et al., 2005).

Because statins impact the early phase of the mevalonate pathway, the potential therapeutic benefits of statin therapy may hinder cancer development by blocking cholesterol synthesis or preventing the production of isoprenoids (non-steroid branch). In addition, statins are known to have anti-inflammatory effects that may also interfere with tumor growth and development.

Suppressors of the mevalonate pathway also include the diverse isoprenoids (Mo and Elson, 2004), mevalonate-derived secondary metabolites of plants (Bach, 1995). The potencies of isoprenoids in suppressing hepatic HMG-CoA reductase activity was found to be strongly correlated to their potencies in tumor suppression (Elson and Qureshi, 1995). Most prominently, the tocotrienols, vitamin E molecules and “mixed isoprenoids” with a farnesol side chain, down-regulate HMG-CoA reductase activity in tumors and consequently, induce cell cycle arrest and apoptosis (Mo and Elfakhani, 2013). Growth-suppressive effect of tocotrienols was attenuated by supplemental mevalonate (Hussein and Mo, 2009).

#### Cholesterol accumulation in lipid rafts

The role of cholesterol in the plasma membranes is complex as it extends beyond the modulation of the fluidity and permeability of the bilayer. Cholesterol is also known to accumulate in specific regions of the membrane, combined with sphingolipids creating small, compartmentalized, and highly stable micro-domains known as lipid-rafts. Lipid-rafts are referred to as the sites of signaling platforms while their specific structure and function depend on their respective lipid compositions and the target proteins involved (Babina et al., 2011). Several proteins that associate with lipid-rafts have been implicated in key signaling pathways associated with malignant progression.

***Apoptosis and cell cycle signaling*** Other than the rapid proliferative behavior of cancer cells, carcinogenic events also feature inhibition/down-regulation of apoptotic pathways. Apoptosis is a highly complex process of programmed cell death that involves an energy-dependent cascade of signaling events (Elmore, 2007). Apoptotic pathways can be classified into two main categories: the extrinsic pathway (death receptor pathway) and the intrinsic (mitochondrial pathway; Elmore, 2007; Babina et al., 2011). Interestingly both of these pathways have been shown to be associated with lipid rafts as they regulate apoptotic signaling events by modulating the cholesterol content of specific membrane regions (Li and Park, 2006; Gajate et al., 2009).

Both Fas receptor (FasR) and TNF-related apoptosis-inducing ligand (TRAIL) receptors 1 and 2, two classes of death receptors, are dependent on translocation into lipid rafts to engage in effective signaling (Gajate et al., 2004; Elmore, 2007; Song et al., 2007). By depleting the membrane of cholesterol, both TRAIL and Fas mediated apoptosis was found to be downregulated (Song et al., 2007; Gajate et al., 2009).

Akt is a serine-threonine specific protein kinase that mediates cell survival and growth and its activation (via phosphorylation) has been shown to occur via a cholesterol dependent mechanism (Zhuang et al., 2002; Li and Park, 2006). In agreement with these findings recent analysis of prostate cancer animal models revealed that elevations of plasma cholesterol cause accumulations of cholesterol in lipid rafts and consequently leads to reduced apoptosis and increased tumor growth via Akt signaling (Li and Park, 2006; Adam et al., 2007; Llaverias et al., 2011; Pelton et al., 2012). In addition, increasing cholesterol levels in lipid-rafts appears to enhance Akt signaling both in vitro and *in* vivo and therefore inducing (cancer) cell survival (Zhuang et al., 2005). Recent studies also show that the subpopulation of Akt present in lipid rafts shows significantly higher substrate specificity compared to the normal Akt (Pommier et al., 2010).

***Tumor growth and development*** Several types of cancer cells exhibit up-regulation of growth factor receptors (Normanno et al., 2006). Additional findings suggest that both EGFR and HER 2 (two types of growth factor receptors) are associated to lipid-rafts and that their signaling events are dependent on the cholesterol content of the lipid-rafts (Chen and Resh, 2002). Once again, the disruption of the lipid rafts via depletion of circulating cholesterol levels interferes with the receptor activation and subsequent inhibition of cell growth and development (Chen and Resh, 2002; Adam et al., 2007; Elmore, 2007)

***Metastasis*** The metastatic phenotype of cancer cells is mediated by signaling mechanisms that decrease cell adhesion and promote cell migration. Integrins and cell-surface glycoproteins such as CD44 are essential components of the cell adhesion mechanism. CD44 is an adhesion molecule expressed in cancer cells, associated with lipid rafts (Oliferenko et al., 1999; Murai, 2012). The modulation of cholesterol either by disruption of lipid rafts or by diminishing plasma levels via statin therapy enhances dissociation of CD44 from the lipid rafts (Murai, 2012) suggesting that cholesterol (membrane or circulating levels) may impact the progression of metastasis.

#### Cholesterol as a precursor of steroidogenesis

The five different classes of steroid hormones are synthesized from a common precursor molecule – cholesterol. The ability of human cells to synthesize steroid hormones is limited to specialized organs, the adrenal cortex and the gonads. Steroid hormones have long been recognized as regulators of cell proliferation and differentiation and are intimately associated with the etiology of breast and prostate cancers. Androgen deprivation therapy is routinely employed in prostate cancer therapy. Recently, it has been shown that prostate cancer cells are able to carry out intracellular synthesis of androgens using cholesterol as the main precursor (Dillard et al., 2008; Locke et al., 2008). Considering that the levels of androgen synthesized by prostate cancer cells seem to be sufficient to activate the androgen receptor (AR), the acquisition of this capacity is thought to be the fundamental mechanism in the development of the androgen resistant prostate cancer phenotype. In a recent report cholesterol has been shown to be an essential precursor for the steroid synthesis process and as a pathway agonist, facilitating the up-regulation of steroidogenic gene expression. These findings are based on the assessment of steroidogenesis enzymes (CYP17A) that are directly correlated with the intra-tumoral cholesterol levels (Mostaghel et al., 2012).

#### Cholesterol as mediator of inflammation

The oxidation and deposition of cholesterol on blood vessels is a common cause of inflammatory response that leads to cardiovascular sequelae. The role of oxidized cholesterol in promoting inflammatory responses could be a factor in subsequent events, including the initiation of carcinogenesis (Pelton et al., 2012). The oxidation of cholesterol occurs via enzymatic reactions or by direct interaction with reactive oxygen species (ROS) producing oxysterols that play important roles in the regulation of bile acids and steroid hormones synthesis (Dufour et al., 2012). Elevated concentrations of oxysterols have been associated with colon, lung, breast, skin, and bile duct cancers (Dufour et al., 2012; Silvente-Poirot and Poirot, 2012a), while hypocholesterolemia decreases the likelihood of oxidation and prostatic inflammation (one of the etiological factors of prostate cancer; Kim et al., 2012).

### CHOLESTEROL TRANSPORT

Cholesterol is a highly insoluble molecule that is transported in the circulation via endogenous transporters known as lipoproteins. Lipoproteins mediate the processing and delivery of dietary cholesterol to peripheral tissues and help maintaining the homeostatic balance by removing the excess cholesterol from peripheral tissues to the liver. It is therefore plausible that lipoproteins play a fundamental role in cancer progression via supplying malignant cells and tumors with cholesterol (Silvente-Poirot and Poirot, 2012b).

A role for lipoproteins in the promotion of cancer progression was initially proposed by a number of investigators (Ho et al., 1978; Vitols et al., 1984, 1990). So far several studies reported the reduction in the levels of plasma lipoprotein components in cancer patients. Originally these changes were described as risk factor for cancer and hypercholesterolemia was thought to contribute to cancer development and progression. Subsequently, an alternate and more plausible rationale has emerged identifying the lowering of plasma cholesterol values as a consequence of malignant tumor growth and development that may actually be utilized as marker for screening undiagnosed cancers (Solomon and Freeman, 2011). Furthermore, it has also been shown that upon successful remission blood cholesterol levels were restored to normal values in cancer patients (Niendorf et al., 1995). When analyzing the lipid profile of cancer patients among the two major cholesterol transporters (HDL and LDL), HDL levels have consistently been shown to be the most affected in malignant tumor development (Muntoni et al., 2009).

During periods of rapid growth and development, Low-density lipoprotein (LDL) particles provide cholesterol to most peripheral tissues via the LDL-receptor, an endocytotic process involving the uptake of the whole lipoprotein particle (Brown and Goldstein, 1986). HDL particles, on the other hand play an important role in removing the excess cholesterol from peripheral tissues, to initiate reverse transport of cholesterol to the liver. The HDL mediated reverse cholesterol transport process is essential to facilitate the liver’s unique ability to process and promote the excretion of excess cholesterol from the body (Simons and Ikonen, 2000). The ABCA1 transporter facilitates this process promoting the efflux of cholesterol from peripheral tissues for incorporation into HDL particles to allow the removal of peripheral cholesterol via lipoprotein receptors to the liver.

While there is a negative correlation between HDL-cholesterol levels and cancer risk, plasma cholesterol levels have been positively correlated with increased expression of cyclin D1, a marker associated with tumor initiation and progression (Llaverias et al., 2011). Several additional studies have shown that lipoproteins are capable of stimulating growth of breast cancer cells in vitro, in addition to enhancing the aggressiveness of malignant tumors in mouse models (Danilo and Frank, 2012). Earlier data showed that, addition of HDL to cell cultures increased the proliferation of human breast cancer cells (Uda et al., 2011). These findings are consistent with several reports on overexpression of the scavenger receptor class B type 1 (SR-B1) receptor in cancer cells, which suggest that during the process of carcinogenesis, tumor cells exploit the HDL mediated removal of cholesterol from peripheral tissues to satisfy their increased cholesterol requirements.

## TARGETING CHOLESTEROL PATHWAYS AS CANCER THERAPY

Accumulating evidence suggests that cholesterol plays an important role in cancer progression and development. Accordingly, several strategies involving regulatory factors that impact cell and blood cholesterol levels have been studied. The inhibition of the mevalonate pathway using statin drugs or biophosphonates result in inhibition of tumor growth and proliferation (Clendening and Penn, 2012; Thurnher et al., 2012). Parallel to the statins and bisphosphonates, isoprenoids (Mo and Elson, 2006) and particularly tocotrienols suppress tumor growth *in vitro* and *in vivo* (Mo and Elfakhani, 2013) consequent to mevalonate deprivation.

Similarly, a drug (ezitimibe), known to inhibit cholesterol absorption by blocking NPC1L1 gut transporters, was shown to interfere with carcinogenesis (Solomon et al., 2009). In this study the administration of ezitimibe significantly decreased tumor-associated blood vessel development, apparently via increased levels of TSP-1, a potent inhibitor of angiogenesis (Solomon et al., 2009). These and other findings suggest that decreasing plasma cholesterol levels may be a plausible therapeutic approach to cancer therapy. Alternate therapeutic strategies have also been proposed attempting to take advantage of the high cholesterol requirements of cancer cells. This strategy relies on “Trojan horse” approach (Lacko et al., 2007) to deliver anti-cancer agents to cancer cells and tumors via receptor-mediated mechanism as discussed below.

## LIPOPROTEINS AS DELIVERY VEHICLES IN CANCER THERAPEUTICS

Lipoproteins share a common structural configuration of a phospholipid monolayer stabilized by a protein meshwork, creating a stable, sealed inner hydrophobic core. Based on this architecture, lipoproteins are ideally suited to accommodate and transport lipophilic compounds, including anti-cancer drugs. This capability of natural and synthetic lipoprotein structures is a major advantage for drug delivery, as it allows a stable hydrophobic environment to accommodate anti-cancer agents and protect them from rapid removal from the blood circulation (Counsell and Pohland, 1982; Bijsterbosch and van Berkel, 1990; Bijsterbosch et al., 1994; Firestone, 1994).

Furthermore, the analysis of several cancer tissues and cancer cells revealed the over-expression of specific components of cholesterol transport, including the SR-B1 (HDL) receptor and the LDL-receptor (Lacko et al., 2002; Tatidis et al., 2002; Llaverias et al., 2011; Gorin et al., 2012). The data showing elevated SR-B1 expression levels in cancer cells and tumors, combined with the finding that HDL-cholesterol levels are lower in cancer patients (compared to normal subjects), suggest that HDL particles are the major suppliers of cholesterol to cancer cells. Based on the SR-B1 receptor mediated drug delivery concept, our laboratory has developed synthetic/reconstituted HDL (rHDL) as a drug delivery platform for anti-cancer agents (Lacko et al., 2006, 2002; McConathy et al., 2008; Mooberry et al., 2009; Shahzad et al., 2011; Sabnis et al., 2012; Sabnis and Lacko, 2012). These drug containing rHDL nanoparticles provide a targeted delivery vehicle with broad therapeutic potential due to the limited expression of the SR-B1 in normal tissue and the over-expression of this receptor by the vast majority of cancer types (Shahzad et al., 2011). The rHDL drug delivery approach is anticipated to markedly suppress tumor growth and to limit or eliminate the off target toxicity of drugs used in cancer chemotherapy (Lacko et al., 2007; Sabnis and Lacko, 2012).

The rHDL delivery of anti-cancer drugs has another potential advantage associated with the selective uptake mechanism of the SR-B1 receptor that facilitates the cytoplasmic delivery of rHDL core components without internalization of the lipoprotein particle (Mooberry et al., 2009; Ng et al., 2011). This mechanism may be of particular interest as it avoids the lysozomal environment, characteristic of the endocytotic pathway, frequent cause of drug inactivation (Rensen et al., 2001).

## CONCLUSIONS

While the impact of cholesterol on oncogenic events, including tumor development, cell migration and angiogenesis have been recognized (Buchwald, 1992; Mo and Elson, 2006, 2008), the mechanisms underlying the role of cholesterol in these events is yet to be fully elucidated. The assumption that cholesterol is primarily required for the high proliferative behavior of malignant cells is likely to be too restrictive as cholesterol has also been shown to be essential for the assembly and function of lipid rafts and related signaling pathways that mediate carcinogenesis. In addition, cholesterol is known to serve as a precursor for steroid hormone synthesis, promote cell migration and mediate of inflammatory process, a key contributor to carcinogenesis. Consequently, targeting cholesterol and lipoprotein pathways is a potentially powerful strategy for cancer therapy. Several studies have explored the inhibition of cholesterol synthesis via the mevalonate pathway as a therapeutic approach for cancer therapy (Buchwald, 1992). While some of the evidence from these studies suggests that statin drugs inhibit carcinogenesis, currently there is no consensus regarding the long-term clinical benefits of this approach at least partially due to their dose-limiting toxicities (Thibault and Samid, 1996).

Utilizing lipoprotein transport for the delivery of anti-cancer agents to cancer cells and tumors is a promising therapeutic approach (Ng et al., 2011; Sabnis and Lacko, 2012). Important features of this concept are the targeted selective tumor delivery of anti-cancer drugs and the reduction of peripheral toxicity to normal cells (Ng et al., 2011; Sabnis et al., 2012, 2013; Sabnis and Lacko, 2012). Overall, in our view, the thorough exploration of the mechanism(s) whereby cholesterol may impact carcinogenesis and/or metastasis is a key research topic that should be vigorously pursued in order to enhance the effectiveness of the current state of cancer therapeutics.

## Conflict of Interest Statement

Drs. Andras G. Lacko, Walter J. McConathy and Nirupama Sabnis are affiliated with LipoMedics, a startup biotech firm interested in developing enhanced formulations for the systemic delivery of anti-cancer drugs.
